# Quantifying how single dose Ad26.COV2.S vaccine efficacy depends on Spike sequence features

**DOI:** 10.21203/rs.3.rs-2743022/v1

**Published:** 2023-05-31

**Authors:** Craig Magaret, Li Li, Allan deCamp, Morgane Rolland, Michal Juraska, Brian Williamson, James Ludwig, Cindy Molitor, David Benkeser, Alex Luedtke, Brian Simpkins, Lindsay Carpp, Hongjun Bai, Bethany Deariove, Alexander Greninger, Pavitra Roychoudhury, Jerald Sadoff, Glenda Gray, Sanne Roels, An Vandebosch, Daniel Stieh, Mathieu Le Gars, Johan Vingerhoets, Beatriz Grinsztejn, Paul Goepfert, Carla Truyers, Ilse Van Dromme, Edith Swann, Mary Marovich, Dean Follmann, Kathleen Neuzil, Lawrence Corey, Ollivier Hyrien, Leonardo Paiva de Sousa, Martin Casapia, Marcelo Losso, Susan Little, Aditya Gaur, Linda-Gail Bekker, Nigel Garrett, Fei Heng, Yanqing Sun, Peter Gilbert

**Affiliations:** Fred Hutchinson Cancer Center; Fred Hutchinson Cancer Center; Fred Hutchinson Cancer Center; MHRP-HJF; Fred Hutchinson Cancer Center; Kaiser Permanente Washington Health Research Institute; Fred Hutchinson Cancer Center; Fred Hutchinson Cancer Center; Emory; University of Washington; Pitzer College; Fred Hutchinson Cancer Center; WRAIR; Walter Reed Army Institute of Research; University of Washington; University of Washington; Janssen Research & Development, LLC; South African Medical Research Council; Janssen R&D; Janssen R&D; Janssen Vaccines & Prevention BV; Janssen Vaccines and Prevention B.V.; Janssen Pharmaceutica N.V., Beerse, Belgium; Evandro Chagas National Institute of Infectious Diseases-Fundacao Oswaldo Cruz; Department of Medicine, Division of Infectious Diseases, University of Alabama at Birmingham; Janssen Pharmaceutica N.V., Beerse, Belgium; Janssen R&D, a division of Janssen Pharmaceutica NV; NIAID/NIH; National Institute of Allergy and Infectious Diseases; National Institutes of Health; University of Maryland School of Medicine; Fred Hutchinson Cancer Center; Fred Hutchinson Cancer Research Center; Evandro Chagas National Institute of Infectious Diseases-Fundacao Oswaldo Cruz; Asociación Civil Selva Amazónica; Hospital General de Agudos José María Ramos Mejia; Department of Medicine, University of California, San Diego, CA 92903; St. Jude Children’s Research Hospital; Desmond Tutu HIV centre; Centre for the AIDS Program of Research in South Africa (CAPRISA), University of KwaZulu-Natal, Durban, South Africa 4041; University of North Florida; University of North Carolina at Charlotte; Fred Hutchinson Cancer Center

**Keywords:** Antibody-epitope escape score, COVID-19 vaccine, ENSEMBLE trial, genetic distance, Hamming distance, neutralization resistance, SARS-CoV-2, sieve analysis, vaccine efficacy, viral variants

## Abstract

It is of interest to pinpoint SARS-CoV-2 sequence features defining vaccine resistance. In the ENSEMBLE randomized, placebo-controlled phase 3 trial, estimated single-dose Ad26.COV2.S vaccine efficacy (VE) was 56% against moderate to severe–critical COVID-19. SARS-CoV-2 Spike sequences were measured from 484 vaccine and 1,067 placebo recipients who acquired COVID-19 during the trial. In Latin America, where Spike diversity was greatest, VE was significantly lower against Lambda than against Reference and against all non-Lambda variants [family-wise error rate (FWER) p < 0.05]. VE also differed by residue match vs. mismatch to the vaccine-strain residue at 16 amino acid positions (4 FWER p < 0.05; 12 q-value ≤ 0.20). VE significantly decreased with physicochemical-weighted Hamming distance to the vaccine-strain sequence for Spike, receptor-binding domain, N-terminal domain, and S1 (FWER p < 0.001); differed (FWER ≤ 0.05) by distance to the vaccine strain measured by 9 different antibody-epitope escape scores and by 4 NTD neutralization-impacting features; and decreased (p = 0.011) with neutralization resistance level to vaccine recipient sera. VE against severe–critical COVID-19 was stable across most sequence features but lower against viruses with greatest distances. These results help map antigenic specificity of in vivo vaccine protection.

Initial SARS-CoV-2 vaccine candidates were based on the virus’s original lineage, as represented by the Wuhan-Hu-1 index strain with Spike D614 (NC_045512). As the virus has evolved,^1–4^ efficacy of these vaccines against symptomatic infection has waned,^5,6^ and new vaccine inserts have been developed.

Based on data from a randomized, placebo-controlled vaccine efficacy (VE) trial on clinical outcomes and pathogen sequences isolated from participants experiencing clinical outcomes, sieve analysis assesses how VE depends on pathogen sequence features.^7,8^ Pajon et al.^9^ and Sadoff et al.^10^ showed how the VE against symptomatic COVID-19 was lower against certain variants than against the Reference strain in the phase 3 COVE trial of two doses of Moderna’s mRNA-1273 vaccine and the phase 3 ENSEMBLE trial of a single dose of Janssen’s Ad26.COV2.S vaccine, respectively. [As in ref.^10^, *Reference is* defined as the basal outbreak lineage B.1, which bears the D614G mutation.] Cao et al. showed that VE was higher in COVID-19 VE trials where circulating viruses had shorter Spike sequence Hamming distances to the vaccine strain.^11^ These sieve analyses only considered Spike viral variation defined by the WHO-defined variant category or the unweighted Spike protein distance. They did not assess how VE depends on other Spike sequence features, such as at the level of individual mutations or features that impact immunological functions such as anti-SARS-CoV-2 neutralization,^12–17^ relevant given the strong evidence of neutralizing antibodies as a cross-platform correlate of protection.^18–20^

We report here the results of a sieve analysis of the ENSEMBLE trial, which enrolled over 40,000 participants and was conducted in the US, South Africa, and six countries in Latin America. The sieve analysis considers baseline SARS-CoV-2 seronegative per-protocol participants and the primary endpoint (moderate to severe–critical COVID-19), as well as the severe–critical COVID-19 endpoint, during the double-blinded period of follow-up. We focus the main text on the Latin America results given the greatest information for sieve analysis as noted below.

## Results

### SARS-CoV-2 sequence data

**Figure 1:**
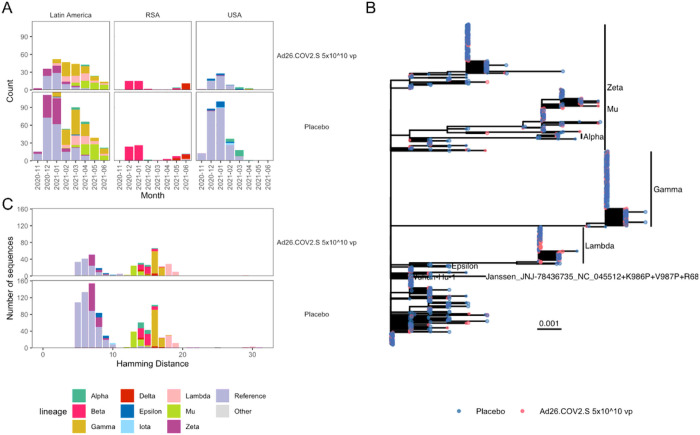
Circulating SARS-CoV-2 lineages in Latin America have greater diversity than in South Africa or the United States. (A) The distribution of SARS-CoV-2 lineages of COVID-19 primary endpoints. The number of lineage sequences identified each month is shown for vaccine and placebo participants. (B) A phylogenetic tree based on the amino acid sequences from Latin America for the Spike protein. Tips are colored to indicate vaccine (red) or placebo (blue). (C) The distribution of variant sequences identified in Latin America as a function of their Spike Hamming distance from the vaccine insert.

A total of 1,345 SARS-CoV-2 Spike amino acid sequences were obtained from 1,224 participants experiencing the moderate to severe-critical primary endpoint. All sequences were variant-typed to either the Reference lineage or to one of nine different WHO-defined variants (Fig. 1A) (**Table S5**). Lineages that circulated at the beginning of the study period, e.g., Reference, were closer to the sequence from the vaccine insert than later emerging lineages, with Lambda the most distant (Fig. 1B–C).

### Greater SARS-CoV-2 Spike diversity in Latin America than in South Africa and the US

Most sequences were obtained from participants in Latin America (n = 776) with additional sequences from the US (n = 323) and South Africa (n = 125) (**Table S6**). Five main variants circulated in Latin America (Reference, Zeta, Gamma, Lambda, Mu), while the South African sequences were 76% Beta and 17% Delta, and the US sequences were 85% Reference (Fig. 1A). There was greater Spike AA sequence diversity in Latin America compared to South Africa and the US (Rao’s Q = 10.1 vs. 7.7 vs. 3.3, respectively; **Fig. S1**).

The succession of distinct co-circulating variants in Latin America and the resulting broadest dynamic range of inter-individual sequence diversity, and the greatest number of COVID-19 endpoints, implies that sieve analyses of the Latin America region have the greatest statistical power. In contrast, the domination of the Reference lineage in the US and the Beta and Delta lineages in South Africa constrained the sequence diversity’s dynamic range and limited the power of these sieve analyses. Therefore, we focus on the results from Latin America, with the US and South Africa results reported in the Supplementary Materials.

### Differential vaccine efficacy against COVID-19 by SARS-CoV-2 lineage

**Figure 2:**
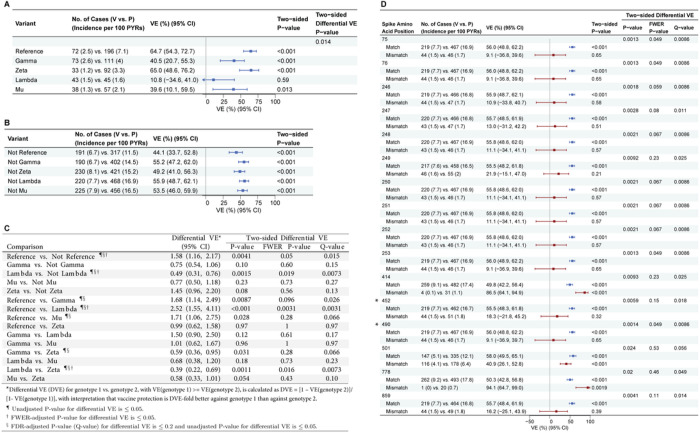
For the Latin America cohort, (A) vaccine efficacy (VE) estimates against the primary COVID-19 endpoint caused by SARS-CoV-2 lineages (lineage “X”); (B) VE estimates against the primary COVID-19 endpoint caused by all other lineages combined (“Not X”); (C) differential VE estimates against the primary COVID-19 endpoint across pairs of lineages or across a lineage (“X”) vs. all other lineages (“Not X”); and (D) VE estimates against the primary COVID-19 endpoint caused by SARS-CoV-2 with a vaccine-matched or vaccine-mismatched residue at each of the 16 spike amino acid residues with differential VE (q-value < 0.2 and unadjusted p ≤ 0.05). Results for VE against matched residue genotypes are shown in blue and for mismatched residue genotypes in maroon. In (**D**), the two amino acid positions hypothesized to impact VE (452 and 490) ^24,26,27^ are identified with an asterisk. For each geographic-region analysis, lineages with at least 20 COVID-19 endpoints were included, and amino acid positions with at least 20 vaccine-mismatched COVID-19 endpoints were included. CI, confidence interval.

All reported results on VE by SARS-CoV-2 features are based on feature-specific proportional-hazards models^21,22^ (see the SAP). Figure 2A shows VE against the primary COVID-19 endpoint caused by the Reference, Gamma, Zeta, Lambda, and Mu lineages, and Fig. 2B shows VE against the primary COVID-19 endpoint caused by the groupings of all other lineages excluding each individual lineage (“not-lineage”). Figure 2C shows differential VE against pairs of lineages or against pairs of lineage vs. not-lineage. VE was significantly higher against Reference than against Lambda and against not-Reference lineages [family-wise error rate (FWER) p < 0.05]. It was also significantly higher against not-Lambda vs. Lambda and against Zeta vs. Lambda (FWER p ≤ 0.05), and higher against Reference vs. Gamma, Reference vs. Mu, Zeta vs. Gamma, and Zeta vs. Mu (q-value ≤ 0.20).

### Vaccine efficacy greater against COVID-19 caused by SARS-CoV-2 genotypes defined by individual Spike AA position residues matching the vaccine strain

We scanned across all Spike AA positions with sufficient residue variability (at least 20 endpoints with a vaccine-mismatched residue: n = 37 positions). VE significantly differed (q-value ≤ 0.20) by residue match vs. mismatch to the vaccine strain residue at 16 positions (Fig. 2D; 4 positions with FWER p ≤ 0.05: 75, 76, 253, 490). Similarly, when assessing the presence or absence of specific residues at each AA position, VE significantly differed (q-value ≤ 0.20) for 38 residues (75V vs. not-75V and 76I vs. not-76I with FWER p ≤ 0.05) at the same 16 positions. **Figure S4** shows the distributions of residues at these 16 positions. Thirteen of these 16 AA sites (Fig. 2D) were sites harboring characteristic mutations of the Lambda variant and not for any other variants, and very highly covaried with Lambda vs. not-Lambda (**Fig. S5**, Mstar^23^ > 0.85), thereby providing nearly equivalent signatures of differential VE captured by Lambda vs. not-Lambda. The full results of the covariability analysis are in the Supplementary Materials.

Four of the 1277 analyzed Spike positions (417, 452, 484, 490) were pre-specified as being hypothesized to impact neutralization based on an association with a reduced neutralizing antibody response in mRNA vaccine recipients,^24–26^ or evidence for increased transmissibility (452)^24^ or increased infectivity in vitro (452, 490).^24,26,27^ Of these sites, positions 452 and 490 were found to significantly impact VE (FWER p ≤ 0.05).

**Figures S2B, S3B**, and **S6** provide complete results including by geographic region.

### Vaccine efficacy against COVID-19 decreases with increasing protein distance to the vaccine-strain in Spike, receptor-binding domain (RBD), N-terminal domain (NTD), and S1

**Figure 3:**
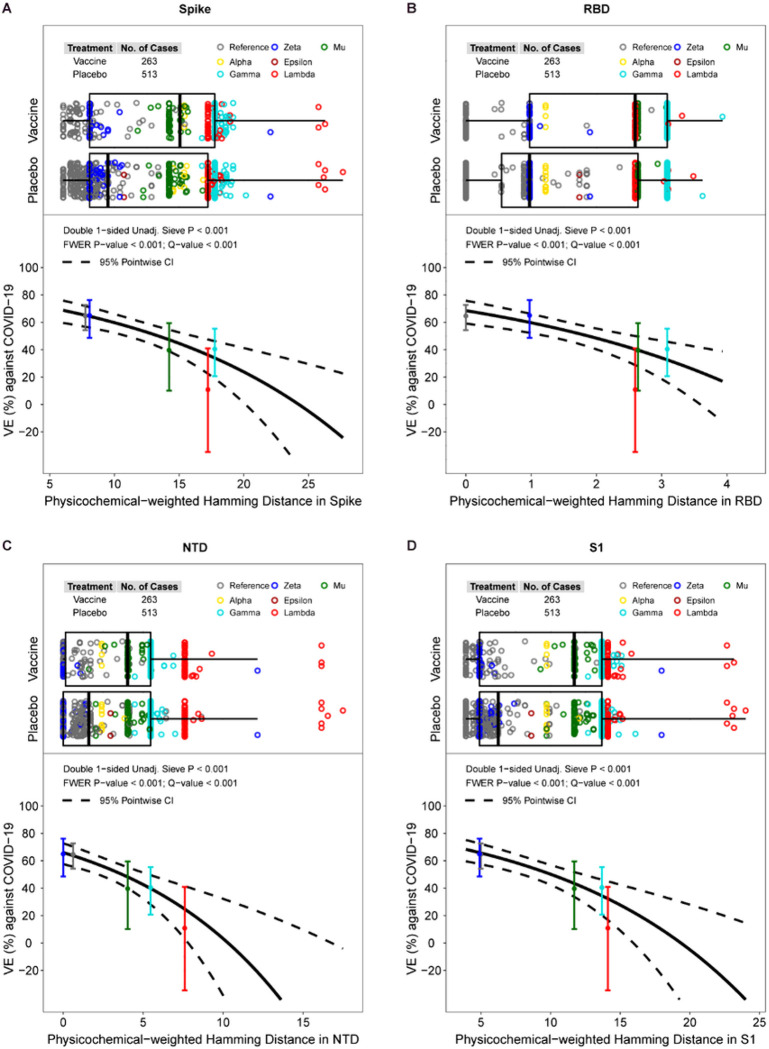
For the Latin America cohort, vaccine efficacy (VE) against the primary COVID-19 endpoint by physicochemical-weighted Hamming distances in (A) Spike, (B) the RBD domain, (3) the NTD domain, or (4) the S1 region of the disease-causing SARS-CoV-2 isolate to that of the vaccine-insert sequence. The top plot in each panel shows the distributions of distances by treatment arm, color-coded by lineage. The bottom plot in each panel shows the estimated VE by SARS-CoV-2 sequence distance. The dotted lines are pointwise 95% confidence intervals. The dots are overall VE estimates for the given lineage placed at the lineage-specific median distance of placebo arm endpoints, with vertical bars indicating their pointwise 95% confidence intervals. Two Zeta sequences are visible outliers from other Zeta sequences; both sequences have two large deletions (9AA and 7AA in length) in the NTD. The plots reveal that Lambda has two sub-lineages, one (n = 79) with range of distances 17.2–18.9 and a second (n = 9) with range of distances 25.8–27.7, due to a 13-AA deletion between sites 64 and 76.

VE significantly decreased with physicochemical-weighted Hamming distance (between the observed vs. vaccine insert sequence) for Spike, RBD, NTD, and S1 (Fig. 3, FWER p < 0.001) but not for S2 (p = 0.78). Against viruses with shortest Spike distances (average 6 residue mismatches), VE was 69% (95% CI: 60–76%), and against viruses with 25th, 50th, 75th, and 95th percentile Spike distances (average 8.1, 12.9, 17.8, 18.6 residue mismatches), VE was 64% (56%, 71%), 52% (44%, 58%), 34% (19%, 46%), and 30% (13%, 44%), respectively. The median distances of sequences for vaccine:placebo were 15.0:9.5 for Spike, 2.6:1.0 for RBD, 4.0:1.6 for NTD, 11.7:6.2 for S1, and 3.1:3.2 for S2. **Tables S7 and S8** show inferences about differences in mean distances of vaccine vs. placebo sequences. **Figs. S7-S11** and **Table S9** provide complete results including by geographic region, where **Table S9** shows that VE decreased with weighted Hamming distance for RBD, NTD, and S1 in the US (q-value ≤ 0.20).

By lineage, ordered by placebo arm COVID-19 endpoint Spike distance to the vaccine strain, Reference viruses had 6.0–17.7 residue mismatches, Zeta 8.1–22.1 mismatches, Epsilon 10.7 mismatches, Mu 12.2–16.8 mismatches, Alpha 14.5–16.8 mismatches, Gamma 16.7–20.2 mismatches, and Lambda 17.2–27.7 mismatches. This ordering of lineages by protein distance matches the ordering of the VE estimates by lineage category, suggesting that overall Spike evolution is a reasonable metric capturing VE decline with variant. The results are generally similarly ordered for the RBD, NTD, and S1 distances (**Fig. S12**).

### Vaccine efficacy against COVID-19 decreases with increasing spike antibody-escape score to the vaccine-strain

Neutralization-relevant RBD features were defined where mutations impact binding in deep mutational scanning (DMS) experiments^28^ (see Supplementary Materials. Escape scores were defined for whole-RBD and for each of 10 epitope-specific clusters of AA sites (see Methods), labeled DMS (whole-RBD) and DMS1 through DMS10. Vaccine efficacy significantly decreased (q-value ≤ 0.20) with each of the DMS, DMS2, DMS6, DMS7, and DMS8 escape scores (FWER p ≤ 0.05) as well as for DMS1, DMS5, DMS9 (q-value ≤ 0.20 and FWER > 0.05) (**Table S12**). **Tables S10 and S11** show inferences about differences in mean escape scores of vaccine vs. placebo sequences.

Alternatively, we defined putative antibody footprint site sets (including whole Spike) based on structures of SARS-CoV-2 in complex with antibodies available from the PDB. Each sequence was assigned an escape score based on a class of epitopes (see Supplementary Materials). These features are referred to as PDB1 through PDB14, with the first 12 clusters in the RBD and PDB13 and PDB14 in the NTD. Vaccine efficacy significantly decreased (q-value ≤ 0.20) with the escape scores for PDB4, PDB7, PDB8, and PDB13 (FWER p ≤ 0.05) as well as for PDB1 and PDB3 (q-value ≤ 0.20 and FWER > 0.05) (**Table S15**). **Tables S13 and S14** show inferences about differences in mean escape scores of vaccine vs. placebo sequences.

**Figure 4:**
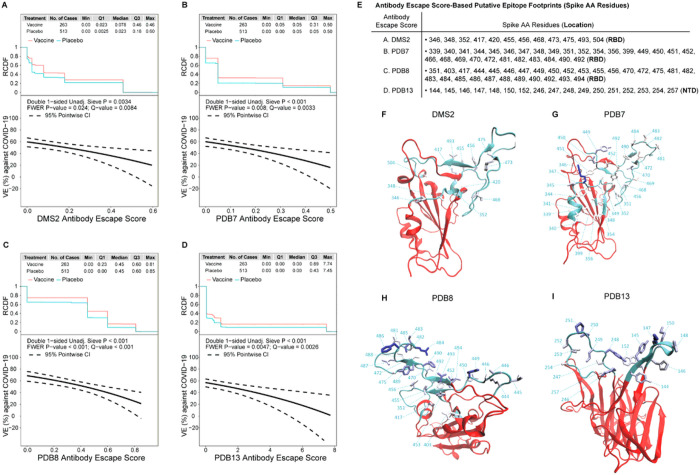
In the Latin America cohort, vaccine efficacy (VE) against the primary COVID-19 endpoint by the SARS-CoV-2 antibody escape score. VE (point estimates as solid line, 95% confidence intervals as dashed lines) is shown by the antibody escape scores for: (**A**) DMS2, (**B**) PDB7, (**C**) PDB8, (**D**) PDB13. The plot at the top of each panel shows the reverse cumulative distribution function (RCDF) of the relevant antibody-binding escape score across SARS-CoV-2 viruses by treatment arm. (**E**) Spike amino acid (AA) residues constituting each antibody escape score-based putative epitope footprint. (**F-I**) For each set of residues constituting an antibody epitope footprint for DMS2, PDB7, PDB8, and PDB13, the image shows the set of AA positions comprising the footprint on a Spike monomer NTD or RBD structure. Cyan ribbons highlight epitope footprint residues while red ribbons make up the rest of RBD [(**F**) DMS2, (**G**) PDB7, and (**H**) PDB8)] or NTD (**I**) (PDB13). Residue numbers and cyan dashed lines are used to label footprint residues. Each structure’s orientation was chosen to best visualize all residues of a footprint. Residues are colored based on their cluster weights going from white to blue with increasing weight.

To interpret the DMS and PDB results, we focus on the epitope-specific features with FWER p ≤ 0.05 that carry the greatest amount of independent information based on inter-correlation and hierarchical clustering analysis (**Supplementary Text, Figs. S13** and **S14**): DMS2, PDB7, PDB8, and PDB13. The sieve analysis results are similar across these four features, with estimated VE at 60–70% against viruses with escape score zero and decreasing to 0%−20% against viruses with maximum escape score. PDB8 and PDB13 rank highest for discriminating VE with slightly greater span of VE point estimates over the range of escape scores (spans 20–60%, 16–60%, 21–69%, and 1–57% for DMS2, PDB7, PDB8, and PDB13, respectively) (Fig. 4A–D). Figure 4E lists the Spike AA residues in each epitope footprint and the visualizations in Fig. 4F–I show the positions comprising the four antibody epitope footprints on a Spike monomer structure. **Figures S15-S23** and **S24-S30** provide complete results for DMS and PDB features, respectively. Another reason PDB8 was highlighted is its balanced contacts across the whole receptor-binding motif (RBM) whereas the other RBM-specific clusters (PDB1-PDB6) are more tightly grouped within a region of the RBM. Among the non-RBM focusing antibodies (PDB7, PDB9-PDB14), PDB7 and PDB13 correspond to the most accessible sites on Spike in a closed prefusion trimer (**Fig. S31**) and these sites are relatively variable among SARS-CoV-2 sequences.

### Lower vaccine efficacy against COVID-19 with NTD features hypothesized to abrogate neutralization

**Figure 5:**
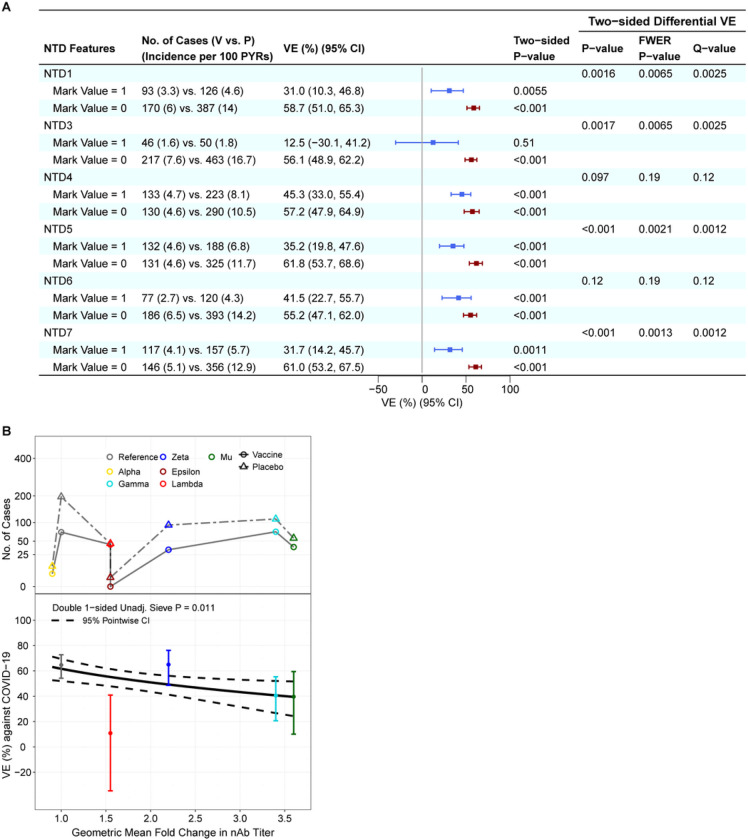
In the Latin America cohort, NTD sequence feature sieve analysis and neutralization phenotype sieve analysis. (A) Vaccine efficacy (VE) estimates against the primary COVID-19 endpoint caused by SARS-CoV-2 with (vs. without) a NTD feature value, screened in as a specific hypothesis-driven neutralizing antibody (nAb) correlate of protection. VE estimates against SARS-CoV-2 harboring the NTD feature value are shown in blue; those against SARS-CoV-2 without the NTD feature value are shown in maroon. (B) VE against the primary COVID-19 endpoint by geometric fold change in neutralizing antibody titer against the disease-causing SARS-CoV-2 variant vs. against the D614G Reference strain. The top plot shows the numbers of cases by treatment arm and color-coded by lineage. The bottom plot shows the estimated vaccine efficacy by geometric fold change in nAb titer against the disease-causing SARS-CoV-2 variant vs. against the D614G Reference strain. The dashed lines are pointwise 95% confidence intervals. The dots are VE point estimates against the given lineage, with the vertical bars showing 95% confidence intervals.

Seven dichotomous NTD features (see Supplementary Materials) were assessed for a sieve effect as for vaccine-match vs. vaccine-mismatch binary features. Six of the 7 NTD features significantly impacted VE (q-value ≤ 0.20): NTD4, NTD6, NTD1, NTD3, NTD5, and NTD7 (where the last four also had FWER p ≤ 0.05) (Fig. 5). **Figure S32** shows the spatial locations in the NTD of the features that impacted VE (FWER p ≤ 0.05).

### Vaccine efficacy greater against lineages with lower variant-neutralization resistance to Ad26.COV2.S vaccine recipient sera

All of the sieve analyses study how VE depends on Spike AA features except one: a neutralization sieve analysis that scores each virus’s lineage by its experimentally measured sensitivity to neutralization by Ad26.COV2.S vaccinee sera.^29,30^ VE decreased with this variant-neutralization resistance score (p = 0.011) (Fig. 5B). Under one model for the neutralization assay being a perfect correlate of protection, the estimates of VE for each of the five lineages would fall on the curve of VE by variant-neutralization resistance score. Lambda had evidence of deviating from the curve, with VE 55% (48, 62%) based on its measured neutralization sensitivity compared to VE 11% (−35, 41 %) based on direct analysis of Lambda ignoring neutralization data. In contrast, the weighted Hamming distance analyses yielded VE estimates at Lambda-variant distance values that are closer to the VE 11% figure.

**Figure S33** provides complete results by geographic region.

### Multivariable virus features as predictors of treatment arm

A variable importance measure analysis by ensemble machine learning^31^ of COVID-19 endpoint cases compared how well AA sequence features predicted treatment arm (results in **Fig. S34** and the **Supplementary Text**).

### Assessing the severe-critical COVID 19 endpoint

Differential VE against severe-critical COVID-19 by lineage could only be assessed for Latin America, with VE of 83% (64, 92%) against Reference, 64% (26, 83%) against Gamma, 94% (−27, 100%) against Zeta, 62% (−31%, 89%) against Lambda, and 84% (42, 96%) against Mu (**Table S16**). There was no evidence of variation in VE across the lineages (p = 0.50) (T**able S16, S17**). The estimates of VE were similar/stable across AA positions with vaccine-matched vs. vaccine-mismatched residue, with all unadjusted p-values for differential VE above 0.05 (**Fig. S35**). For the key positions 452 and 490 found to show sieve effects for the primary COVID-19 endpoint, the results for the severe-critical COVID-19 endpoint were VE 79% (68, 87%) against 452-matched virus compared to VE 70% (3, 91%) against 452-mismatched virus (p = 0.58 for difference), and VE 80% (68, 87%) against 490-matched virus compared to VE 62% (−31, 89%) against 490-mismatched virus (p = 0.34 for differential VE). For the DMS antibody escape score distances, the data support stable VE across the distances (**Table S18**). Similarly, the data support stable VE across RBD and PDB Spike-antibody escape scores (**Table S19**). VE was stable by variant-neutralization resistance score, with VE = 84% (67%, 92%) for the most sensitive lineage (ancestral) and VE = 73% (50, 85%) for the least sensitive lineage (Mu) (p = 0.33, **Fig. S36**).

### Vaccine efficacy against severe–critical COVID-19 decreases with increasing protein distance to the vaccine-strain and by NTD features hypothesized to abrogate neutralization

There was a trend of VE against severe–critical COVID-19 decreasing with the weighted Hamming distance for the Spike, NTD, and S1 regions (q-values = 0.20) (**Table S20, Figs. S37, S39, S40**). The point estimates of VE suggested moderate declines of VE with distances. For example, the VE for Spike was 87% (71%, 94%) against viruses with shortest distance of 6 and 66% (34%, 83%) against viruses with long distance of 20 (p = 0.12). **Figs. S37-S41** and **Table S20** provide complete information by geographic region. In addition, while VE was stable across levels of NTD1 through NTD4 (p > 0.20), it differed by levels of NTD5, NTD6, and NTD7, with VE of 61 % (31,78%) vs. 88% (76, 94%) for the two NTD5 genotypes (q = 0.10 for difference), VE of 60% (20, 80%) vs. 84% (72, 91%) for the two NTD6 genotypes (q = 0.12 for difference), and VE of 64% (32, 80%) vs. 85% (73, 92%) for the two NTD7 genotypes (q = 0.12 for difference) (**Table S21**).

## Discussion

Sieve analysis compares genotype-specific or immunophenotype-specific COVID-19 incidence between randomized study groups, therefore directly assessing causal effects of vaccination and providing inferences for how vaccine efficacy depends on SARS-CoV-2 features. In addition to the strength of a randomized, double-blinded placebo-controlled phase 3 trial, the present sieve analysis of ENSEMBLE had ample statistical precision due to the large number of SARS-CoV-2 Spike sequences (measured from more than 1,200 participants) and the broad proteomic variability of the SARS-CoV-2 Spike sequences causing these endpoints. Consequently, the sieve analysis could provide many insights into how the efficacy of the Ad26.COV2.S vaccine, evaluated in baseline SARS-CoV-2 negative individuals, depended on virus features.

In the Latin American cohort, VE against the moderate to severe–critical COVID-19 primary endpoint significantly declined with Spike sequence distance as measured in myriad ways, including lineage, weighted Hamming distances calculated for Spike, RBD, NTD, and S1, scores reflecting degree of escape from epitope-specific antibodies computed using deep mutational scanning or based on crystal structures in the Protein Data Bank (PDB), and NTD features previously shown to impact neutralization. Estimates of VE by lineage were consistently ordered by the distances of the different lineages to the vaccine strain. VE declined similarly with Spike, RBD, NTD, and S1 distances (VE about 70% against viruses closest to the vaccine and 20% against viruses beyond the 90–95th percentile of distances) but did not depend on S2 distances. This may be explained by S2’s relative conservation when compared to S1. As such, almost all variant-characteristic mutations are not in S2, and none of the prescribed antibody epitope footprint clusters included S2 positions (only rare epitopes in PDB mapped to S2), reflecting S2’s ‘stalk’ location and relative lack of exposure to the immune system.

VE significantly declined with 14 of the 20 evaluable antibody epitope escape scores. Six antibody-epitope clusters had no evidence of impacting VE: DMS3, PDB2, PDB5, PDB6, PDB9, PDB14. Of the 14 clusters with a sieve effect, 9 include at least one site that harbors a characteristic mutation of Lambda, whereas 3 include site 417 twhich is a characteristic mutation of Mu and Gamma, 1 includes site 501 that harbors a characteristic mutation of Gamma, Alpha, and Mu, and 1 includes both sites 417 and 501. Thus the 9 sieve-effect clusters appear to be driven by the differential VE by Lambda vs. not-Lambda, whereas the other 5 appear to be driven by mutations at the important sieve-effect sites 417 and 501 that impact neutralization. Of the 6 non-sieve-effect clusters, only one (PDB14) included a site harboring a characteristic mutation of Lambda, site 75, which was a sieve-effect site with FWER p ≤ 0.05. The potential for sieve effects in different epitope sets depends on many factors including level of accessibility to neutralizing antibodies, conservation, and the narrowness of the footprints on the tridimensional structure they target (**Fig. S31**).

Neutralizing antibody assays have performed well at predicting vaccine efficacy against COVID-19 and severe-critical COVID-19 across SARS-CoV-2 lineages.^19,20,32^ Importantly, one of the sieve analyses in the present work scored viruses by their lineage’s directly measured resistance to neutralization by sera from ENSEMBLE Ad26.COV2.S vaccine recipients, providing a way to study a neutralization correlate of protection (CoP) in a complementary way to individual-level and population-level immune correlates analyses.^33–35^ VE significantly declined against lineages with greater neutralization resistance scores, providing validation of pseudovirus neutralization titer as a CoP However, the lineage scores were estimated from only eight ENSEMBLE vaccine recipients, albeit the scores are supported by additional data from 17 Ad26.COV2.S vaccine recipients in the COV2001 phase 1/2a study.^36^

The relative prevalence of SARS-CoV-2 lineages changed over time (Fig. 1A and Fig. 1 of ref.^10^) where in Latin America the median (range) number of days from enrollment until the COVID-19 endpoint among placebo recipients was 48 (15, 197) for Reference, 45 (15, 141) for Zeta, 114 (42, 220) for Gamma, 126 (57, 204) for Lambda, and 170 (109, 219) for Mu. If newer variants tended to expose participants later in follow-up than older variants it could cause spurious genotypic sieve effects that are instead due to waning vaccine efficacy. This potential bias was mitigated by controlling for calendar time of enrollment in the sieve analyses.

The Ad26.COV2.S vaccine sieve effects observed here, based on data collected prior to July 10, 2021, revealed broader vaccine adaptation features as several sieve signature sites showed mutations in subsequent variant waves. Hence, mutations at sites 452, 484 and 501 are dominant in currently circulating Omicron sub-lineages [global proportion between 2022–12-04 and 2022–12-10: L452R = 87.2%, E484A = 98.5%, N501Y = 99.2%^37^]. While the sieve signature F490S had been rare until the end of 2022, this mutation became dominant in early 2023 with the global rapid spread of XBB.1.5 variants. The fact that sieve analysis predicted currently relevant mutations could be expected since SARS-CoV-2 has shown remarkable patterns of convergent evolution since the initial appearance of variants, with numerous recurrent mutations, especially in the RBD, shared across lineages over time.^38^

A strength of this study was it was conducted in three separate geographic regions with different circulating lineages, which contribute insights based on these lineages and their characteristic signature mutations, and different distributions of genetic distances of circulating sequences to the vaccine strain. The analyses of Latin American study sites provided the greatest insights given that 63% of primary COVID-19 endpoints with sequence data were in Latin America where the circulating SARS-CoV-2 sequences were the most diversified. All features showing sieve effects in the US also showed sieve effects in Latin America, constituting independent replication of results. The result of no sieve effects in South African study sites can likely be explained by the vast majority of circulating sequences being Beta or Delta variants with limited dynamic range of genetic distances within each variant and a lack of Reference viruses that are close to the vaccine strain.

Another strength of this study was that VE against severe-critical COVID-19 could be assessed. The results support that VE against this endpoint also declines with Spike sequence distance as measured in multiple ways, yet with VE starting higher against viruses closest to the vaccine strain and diminishing less rapidly with increasing degrees of sequence mismatch. Overall, the finding that protection against severe-critical COVID-19 is more invariant to sequence changes than against less-symptomatic COVID-19 may have clinical implications for planning updates of vaccines with new variants. The severe-critical classification covers a broad spectrum of clinical phenotypes ranging from individuals with only repeated low partial pressure of oxygen to severe pneumonia requiring respiratory support. Protection against hospitalization with severe consequences is clinically most important but sieve analysis specific to this outcome could not be performed given small numbers of cases. Yet, ENSEMBLE and post-approval trials have shown high Ad26.COV2.S efficacy against this outcome especially in South Africa after a 6-month boost, suggesting that neutralization resistance and sequence variation may be playing a less dominant role in vaccine-induced protection against the most serious disease, perhaps due to CD8 + T cells.^39^

## Methods

### Trial design, study cohort, and COVID-19 endpoints

Trial enrollment began on September 21, 2020. The end of the double-blind period varied by country; the data cutoff for this analysis was July 9, 2021. The main endpoint for sieve analysis is the same COVID-19 primary endpoint (moderate to severe-critical) as in the primary analyses, ^10,40^ restricting to endpoints starting 14 days post vaccination. Sieve analyses were also conducted for severe–critical COVID-19, again using the same definition as used in the primary papers.^10,40^ Analyses were conducted in the per-protocol baseline seronegative cohort.^40^ See Section 1 of the Statistical Analysis Plan (SAP provided in ref.^41^ and as supplementary material) and the Supplementary Materials for further details.

### SARS-CoV-2 sequencing and sequence data

SARS-CoV-2 Spike sequences were generated and variant-typed as described.^40^ Sequences were selected for analysis if they were obtained within 36 days following the first RNA-positive timepoint associated with the first moderate to severe-critical COVID-19 primary endpoint. See the Supplementary Materials for further details.

### Neutralizing antibody titers

Neutralizing antibody titers were measured to a panel of Spike antigens representing the Reference strain B.1.D614G and several variants.^29,30^ Each variant was assigned a score defined as the log10-transformed ratio of geometric mean titer of vaccinee sera against the variant and the geometric mean titer of vaccinee sera against the Reference strain.

### Sieve analysis

This analysis was specified in advance and documented in the SAP The sieve analyses were conducted for each of the four geographic regions: Latin America, South Africa, the US, and the three geographic regions pooled (hereafter, ‘geographic-region analyses’). Details on specification of spike amino acid (AA) sequence features for sieve analysis, prospective vaccine efficacy sieve analysis, neutralization hypothesis-driven sieve analysis, and multiple hypothesis testing adjustment for AA sequence sieve analysis are provided in the Supplementary Materials.

Additional details on covariability analysis, quantification of viral diversity, antibody escape scores [deep mutational scanning (DMS) and Protein Data Bank (PDB)], variant-neutralization sensitivity score assigned to variants, handling of missing sequences, and structural modeling is also in the Supplementary Materials.

## Figures and Tables

**Table 1:** Numbers of primary endpoint COVID-19 cases with Spike amino acid sequence data by treatment arm and geographic region. A primary endpoint case is defined as the moderate to severe-critical primary COVID-19 endpoint in the per-protocol baseline seronegative cohort, with disease onset starting 14 days post vaccination through to a participant’s unblinding date.

	Geographic Region							
	Latin America		South Africa		United States		Pooled	
Primary endpoint case lineage	Vaccine (329)^1^	Placebo (634)	Vaccine (62)	Placebo (110)	Vaccine (93)	Placebo (323)	Vaccine (484)	Placebo (1067)
Reference	72	196	1	4	52	221	125	421
Alpha	4	10	1	2	4	16	9	28
Beta	-	-	36	59	-	-	36	59
Delta	-	-	11	10	-	-	11	10
Epsilon	-	2	-	-	8	15	8	17
Gamma	73	111	-	-	1	-	74	111
Iota	-	-	-	-	-	4	0	4
Lambda	43	45	-	1	-	-	43	46
Mu	38	57	-	-	-	-	38	57
Zeta	33	92	-	-	1	1	34	93
No Sequence Obtained	66	121	13	34	27	66	106	221

1 Numbers in parentheses are numbers of moderate to severe-critical COVID-19 primary endpoints caused by the listed SARS-CoV-2 lineage, regardless of availability of SARS-CoV-2 sequence data

## Data Availability

All sequences involved with this study are available on GISAID, including their contributors’ details, such as accession number, virus name, collection date, originating lab, submitting lab and the list of authors. The sequences are available in two groups: the sequences obtained from study participants (Supplementary Data 1) and the sequences curated by LANL to define the canonical variant sequences (GISAID Identifier: EPI_SET_221208yn; doi: 10.55876/gis8.221208yn). Custom code for the structural modeling has been deposited at Zenodo (doi: 10.5281/zenodo.7869358).
